# Exploring the Impact of Workplace Hazing on Deviant Behavior in the Hospitality Sector: The Roles of Emotional Exhaustion and Hope and Optimism

**DOI:** 10.3390/bs15020129

**Published:** 2025-01-25

**Authors:** Osama Aljaier, Ahmad Alzubi, Amir Khadem, Kolawole Iyiola

**Affiliations:** Department of Business Administration, Institute of Graduate Research and Studies, University of Mediterranean Karpasia, Mersin 33001, Turkey; 210634057@std.akun.edu.tr (O.A.); kolawole.iyiola@akun.edu.tr (K.I.)

**Keywords:** workplace hazing, deviant behavior, emotional exhaustion, hope and optimism, transaction of stress theory, hospitality sector

## Abstract

Workplace hazing remains a critical concern in the hospitality sector, known for its high-pressure environments and hierarchical dynamics. Drawing on transactional stress theory, this study explores the intricate relationships between workplace hazing, emotional exhaustion, and deviant behavior, with a focus on the moderating roles of hope and optimism. The research aims to examine how these psychological factors buffer the negative impacts of workplace hazing on employee behavior. Using a quantitative approach, data were collected from 494 valid responses comprising employees and supervisors from four- and five-star hotels in Aqaba, Amman, and Petra, Jordan. Convenience sampling was employed, and the data were analyzed using SPSS 29 and AMOS 26. The findings reveal that workplace hazing significantly predicts emotional exhaustion and deviant behavior, with emotional exhaustion mediating this relationship. Hope and optimism were found to moderate these effects, suggesting employees who maintain an optimistic outlook are better equipped to handle stress, which, in turn, reinforces their positive mindset, ultimately fostering healthier workplace cultures. The study offers both theoretical and practical implications. Theoretically, it extends the transaction theory of stress by integrating emotional exhaustion and deviant behavior. Practically, it suggests that hospitality organizations should focus on mitigating hazing practices, addressing emotional exhaustion through support mechanisms, and fostering positive psychological traits like hope and optimism to enhance employee well-being and minimize deviant behavior.

## 1. Introduction

The hospitality industry has the most reported incidents of hazing and harassment among all sectors but this is not limited to Jordan. Studies and reports from various countries highlight similar issues. For instance, a study (Raza et al., 2024) in Pakistan found that workplace hazing in the hospitality industry increases moral disengagement and organizational deviance. Additionally, (Pearlman & Bordelon, 2022) the U.S. Equal Employment Opportunity Commission reported a significant increase in harassment reports in the hospitality sector following the #MeToo movement. Another study, conducted by the International Labor Organization (ILO) in 2021, found that 75% of employees experienced workplace harassment and felt unsafe at work. Despite these negative reports, hospitality scholars and leaders emphasize the significance of worker welfare for the industry’s growth and support. Although the hospitality industry supports the global economy by creating new jobs and showing incredible development, in particular, the hospitality sector faces more challenging working conditions. These unfavorable situations can exacerbate a hostile work environment, leading to workplace hazing incidents (Anasori et al., 2020; Said & Tanova, 2021).

Workplace hazing is a pervasive issue that involves the initiation of newcomers through degrading behaviors, which can lead to significant negative outcomes for both employees and organizations (Said & Tanova, 2021). Workplace hazing involves repeated and unwelcome actions aimed at humiliating, punishing, and intimidating a target (Sarwar et al., 2021). Unlike general workplace incivility, hazing is characterized by its persistent and targeted nature. This behavior negatively impacts job satisfaction, motivation, and work performance, leading to emotional exhaustion. While there is broad agreement on the harmful consequences of workplace hazing, the literature has detailed the mechanisms through which these outcomes arise (Said & Tanova, 2021). Emotional exhaustion, a state of chronic physical and emotional depletion, is a key component of burnout and can result from continuous stress and excessive job demands. Employees subjected to workplace hazing may experience heightened levels of emotional exhaustion, leading to deviant behavior as a coping mechanism or form of retaliation (Anasori et al., 2020). Understanding the mediating role of emotional exhaustion in this relationship is crucial for developing strategies to mitigate the adverse effects of workplace hazing.

Additionally, this study examines the moderating roles of hope and optimism, central elements of improving well-being, quality of life, and psychological adjustment. They have also shown that hope and optimism can help people adapt to adversity and reduce the risk of developing mental disorders. These traits promote proactive health behaviors, while pessimism is associated with negative health outcomes. These traits can influence how employees appraise and cope with stress, potentially buffering the negative impacts of workplace hazing. Employees with higher levels of hope and optimism may be better equipped to handle the stress associated with hazing, thereby reducing the likelihood of emotional exhaustion and subsequent deviant behavior (Preena, 2021).

Despite extensive research on workplace stress and employee behavior, there is a significant gap in understanding the specific role of hazing in shaping deviant behaviors within the hospitality sector. Workplace hazing and its specific dynamics and implications within the hospitality sector remain unexplored. This sector is characterized by customer-facing roles, irregular hours, and high turnover. While the link between workplace hazing and deviant behavior is acknowledged, there is a lack of research investigating the psychological mechanisms underlying the relationship with emotional exhaustion, a critical stress outcome as a mediator in this study. Additionally, the positive role of positive psychological resources such as hope and optimism in mitigating the effects of hazing remains underexamined. These gaps highlight the need for a comprehensive theoretical framework transaction of stress theory to explore this relationship.

Integrating approaches from the transaction of stress theory framework, we posit that individuals experience stress when they perceive a mismatch between environmental demands and their coping resources. Hazing as a stressor may lead to emotional exhaustion, which can, in turn, increase the likelihood of deviant behavior as employees struggle to cope with the stress. This study aims to explore the impact of workplace hazing on deviant behavior within the hospitality sector, focusing on the roles of emotional exhaustion as a mediator and positive psychological traits such as hope and optimism as a moderator in these dynamics. Addressing this gap, the study aims to fill the gap in understanding the role of workplace hazing in shaping deviant behavior within the hospitality sector, with a focus on the mediating role of emotional exhaustion and the moderating role of hope and optimism. By doing so, it seeks to provide valuable insights for developing strategies to create a healthier and more supportive workplace culture. To achieve the research objectives, the following research questions were formulated:

RQ1: How does workplace hazing impact deviant behavior in the hospitality sector?

RQ2: Does emotional exhaustion mediate the link between workplace hazing and deviant behavior?

RQ3: How do hope and optimism moderate the relationship between workplace hazing and emotional exhaustion?

**Figure 1:**
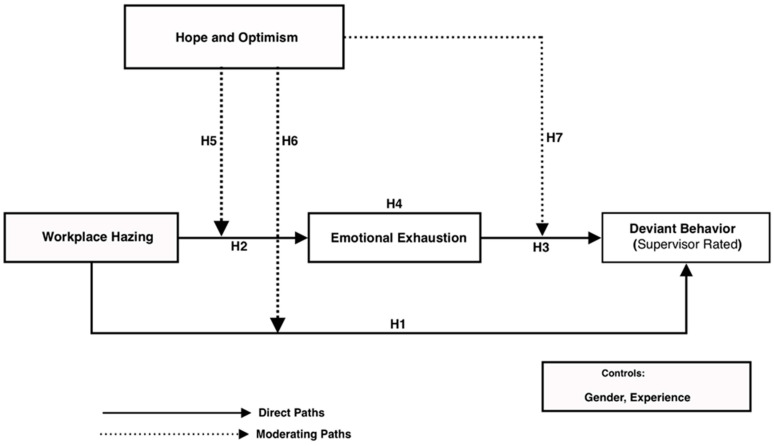
Research Model.

This study aims to contribute to theoretical implications by addressing these research questions. The remaining sections offer a comprehensive literature review and introduce the conceptual framework, highlighting key variables. The proposed research model (Figure 1) formulates and illustrates several hypotheses based on the transaction of stress theory. These hypotheses examine both theoretical and practical implications and effects to investigate the impact of workplace hazing on deviant behavior in the hospitality sector. Section 3 details the research methodology, outlining the methodological processes adopted. Section 4 presents the results, while Section 5 discusses the findings, drawing conclusions and addressing implications, limitations, and future research directions.

## 2. Theoretical Background and Hypotheses Development

### 2.1. Transaction Theory of Stress

The transactional theory of stress, primarily developed by (Lazarus & Folkman, 1984), is often chosen as a preferred model for understanding stress because it emphasizes the dynamic interaction between an individual and their environment, recognizing that a person’s perception and appraisal of a situation significantly influence their stress response, rather than simply attributing stress to external factors alone; this allows for a more nuanced understanding of how individuals cope with stressors and adapt to different situations based on their resources and coping mechanisms. The transaction theory of stress suggests that a person’s ability to manage stress and handle challenges arises from interactions between the individual and their environment. The working environment of the hospitality industry is characterized by irregular and long working hours, role pressure, and work overload. Therefore, hospitality employees facing such stressors may easily become nervous and anxious, in turn, leading to dissatisfaction and negative emotions toward work (Ghorbani, 2023). In the context of stress theory, deviant behavior in the hospitality industry refers to actions that deviate from societal norms and are often considered harmful. These actions can arise as a coping mechanism when individuals experience high levels of stress or strain, leading them to engage in behaviors such as substance abuse, aggression, or withdrawal as a way to manage their negative emotions (Tu et al., 2022).

Connecting the transaction theory of stress, emotional exhaustion refers to a state of depletion and burnout that can occur when someone experiences prolonged or excessive stress. On the other hand, resilience refers to the ability to recover from challenging situations, often facilitated by positive psychological traits like hope and optimism. These traits act as protective factors against the negative effects of stress and promote recovery from emotional exhaustion (Si et al., 2023). In summary, while stress can lead to emotional exhaustion, individuals with strong positive psychological traits like hope and optimism are better equipped to manage stress, bounce back from adversity, and maintain their overall well-being (Vîrgă et al., 2020).

### 2.2. Workplace Hazing

Workplace hazing in the hospitality industry is an issue that often goes underreported due to the hierarchical nature of the industry and the cultural normalization of certain behaviors. Workplace hazing refers to the unofficial, temporary practice of initiating newcomers into their workgroups through degrading behaviors (Mawritz et al., 2022). Despite its prevalence across various cultures and organizations, including those in the hospitality sector, it remains under-researched in both general and hospitality-specific literature. Hazing negatively affects newcomers’ productivity, self-confidence, well-being, and self-esteem, leading to decreased job satisfaction and organizational commitment. In the hospitality industry, hazing can exacerbate existing issues such as hostile behaviors, uncomfortable work environments, and high levels of stress and burnout, ultimately contributing to employee turnover (Ali et al., 2022). These impacts of workplace hazing on deviant behavior within the hospitality sector focus on the roles of emotional exhaustion as a mediator and positive psychological traits such as hope and optimism as a moderator in these dynamics. Using transactional stress theory, workplace hazing in the hospitality industry addresses both the appraisal and coping processes of employees. Organizations can reduce the perception of hazing as a threat by fostering a supportive culture and clearly defining acceptable behaviors through policies and training (Srivastava et al., 2024).

### 2.3. Deviant Behaviour (Supervisor Rated)

Supervisors rate deviant behavior in the hospitality industry as any employee actions that significantly deviate from expected standards of conduct, such as dishonesty, theft, excessive absenteeism, poor work ethic, neglecting customer service, deliberate sabotage, or abusive behavior towards colleagues or guests. These behaviors can negatively impact the business and customer experience, disrupting the smooth operation of the establishment and undermining its reputation (Sarwar et al., 2021). The deviant acts can include a desire to achieve socially desired goals through unconventional means, a feeling of alienation from societal norms, a sense of powerlessness within the social structure, peer pressure, personal psychological issues, a need to rebel against authority, and sometimes even a desire to gain attention or belonging within a subculture that embraces deviant behavior (Tu et al., 2022). Emotional exhaustion, a key mediating factor that can lead to deviant behavior, suggests that when an individual experiences high levels of emotional stress due to overwhelming demands, they are more likely to engage in negative or disruptive actions as a coping mechanism due to their depleted emotional resources. Essentially, being emotionally drained can make someone more prone to deviant behavior (Jia et al., 2022). Transactional stress theory has recognized that positive deviant behaviors can motivate staff and foster a positive work environment (Dula, 2022).

### 2.4. Workplace Hazing and Deviant Behavior

Workplace hazing can lead to deviant behavior through several psychological processes. Chronic stress and emotional exhaustion are key mediators in this relationship. Hazing creates a hostile work environment, leading to prolonged stress and burnout. Employees subjected to hazing may resort to deviant acts as coping mechanisms. For example, forced participation in menial tasks and public ridicule can lead to feelings of helplessness and frustration, prompting deviant behaviors such as theft (Tahir, 2021; Raza et al., 2024). Various industries have highlighted the impact of workplace hazing on deviant behavior. Hazing increases moral disengagement in the hospitality sector, leading to organizational deviance and negative word-of-mouth communication. Additionally, psychological factors such as resilience and social support can mediate the negative effects of hazing. Workplace hazing can have significant organizational consequences and can create divisions within teams, reducing collaboration and trust. Reduced productivity, emotional exhaustion, and stress can lead to decreased job performance and higher absenteeism. Employees subjected to hazing are more likely to leave the organization, leading to higher recruitment and training costs (Ahmed et al., 2024).

According to the transaction theory of stress, cognitive appraisal and coping strategies mediate the dynamic interaction between an individual and their environment, leading to stress. In the context of workplace hazing, employees’ appraisal of hazing as a threat can lead to stress and deviant behavior as a coping mechanism (Lim et al., 2023). Therefore, it is believed that workplace hazing positively correlates with deviant behavior among employees. Previous studies have confirmed the positive link between workplace hazing and deviant behavior (Jiang et al., 2021). In light of the discussion above, the following hypothesis is proposed:

**H1.**  *Workplace hazing is positively related to deviant behavior.*

### 2.5. Workplace Hazing and Emotional Exhaustion

Workplace hazing involves the act of initiating newcomers into their workgroups through degrading behaviors. This practice is common across various industries and can have severe negative impacts on employees and organizations. Workplace hazing can result in emotional exhaustion, a condition characterized by chronic physical and emotional depletion due to continuous stress and excessive personal demands. Burnout’s key component, emotional exhaustion, manifests as feelings of drainedness and overwhelm. In the hospitality industry, a high level of cooperation among employees and an environment that provides social support through emotional, informational, and instrumental resources is necessary (Ali et al., 2020). However, many parts of the world view workplace hazing as a major problem in the hospitality industry, as it damages such cooperation and the organizational environment. Some argue that the hospitality industry is plagued by poor work organization, characterized by high work pressure, low discretion, unregulated managerial control, and the use of vulnerable workers. Increasingly, employees in the hospitality industry are reporting workplace hazing. For instance, an average of 16% of hospitality industry employees report experiencing numerous undesirable acts at their workplace (Teo et al., 2020). The hospitality industry still requires significant efforts to combat workplace hazing.

Job demands and the availability of stress management resources drive responses, building upon the transaction theory of stress. Their mental state and thought patterns influence the behavior of employees. The persistent and targeted nature of hazing can erode employees’ well-being, motivation, and job satisfaction, ultimately leading to reduced job performance and increased turnover rates. Addressing workplace hazing and its consequences is crucial for creating a positive work environment and reducing the incidence of emotional exhaustion (Shaikh & Usmani, 2022). Building on the aforementioned insights, this study formulates the following hypothesis:

**H2.**  *Workplace hazing is positively related to emotional exhaustion.*

### 2.6. Emotional Exhaustion and Deviant Behavior

Emotional exhaustion depletes an individual’s psychological and physical resources, reducing their capacity to adhere to workplace norms. This depletion can lead to deviant behaviors such as absenteeism and interpersonal conflicts. In the hospitality sector, employees experiencing emotional exhaustion may engage in acts like neglecting customer service duties or intentionally damaging property as a form of retaliation (Stein et al., 2021).

Absenteeism. Employees may frequently call in sick to avoid the stressors of the workplace. Interpersonal conflicts and increased irritability and frustration can lead to conflicts with colleagues or supervisors. Kong et al. (2020) highlighted the impact of emotional exhaustion on deviant behavior under modern workplace stressors. For example, the research has shown that high workloads, poor leadership, and organizational changes significantly contribute to emotional exhaustion, which, in turn, leads to deviant behaviors. These findings underscore the importance of addressing these stressors to mitigate their negative effects.

The transaction theory of stress frames emotional exhaustion as a response to stress appraisal. According to this theory, cognitive appraisal and coping strategies mediate the dynamic interaction between an individual and their environment, resulting in stress (Lim et al., 2023). When employees perceive their resources as inadequate to meet the demands of their jobs, they experience stress, which can lead to emotional exhaustion and subsequent deviant behaviors (Kong et al., 2020; Jahanzeb & Fatima, 2017; Jiang et al., 2021). To mitigate the effects of emotional exhaustion, organizations can implement several interventions. Fostering a supportive leadership style can help reduce stress and emotional exhaustion among employees. Encouraging employees to maintain a healthy work–life balance can prevent burnout and emotional exhaustion. Promoting traits like hope and optimism can help employees cope better with stress and reduce the likelihood of deviant behaviors (Kumar & Jin, 2022). From the preceding discussion, the study advances the following hypothesis:

**H3.**  *Emotional exhaustion is positively related to deviant behavior.*

### 2.7. The Mediation Role of Emotional Exhaustion

Emotional exhaustion is characterized by feelings of being expressively overextended and drained, a key component of job burnout (Hobfoll & Ford, 2007). Workplace hazing acts as a stressor that can jeopardize employees’ resources and induce psychological stress. Hazing not only deprives employees of support from their colleagues or superiors but also disrupts their relationships with others (Choi, 2019). Research has shown a positive correlation between workplace hazing and emotional exhaustion, especially in male employees.

The deficiency of work and emotional resources can lead to emotional exhaustion. According to the transactional stress theory (Golparvar et al., 2008), job stress can disrupt employees’ emotional equilibrium, prompting them to show positive behaviors to recover stability. Organizational stressors such as hazing can disrupt employees’ mental stability, potentially leading them to engage in deviant behavior to regain equilibrium. Employees perceive emotional exhaustion as a state of disequilibrium (Hao et al., 2015). Therefore, workplace hazing might push employees into a state of emotional exhaustion, which could then result in deviant behavior. Grounded in the theoretical framework discussed, the study posits the following hypothesis:

**H4.**  *Emotional exhaustion mediates the relationship between workplace hazing and deviant behavior.*

### 2.8. The Moderation Role of Hope and Optimism

Hope and optimism play crucial roles in how employees appraise and respond to stressors, as outlined in the transaction theory of stress. This theory posits that stress is a result of the dynamic interaction between an individual and their environment, mediated by cognitive appraisal and coping strategies. Hope and optimism enable employees to perceive workplace stressors, such as hazing, as challenges rather than threats. This positive appraisal leads to more adaptive coping strategies and lower levels of emotional exhaustion (Duan, 2021).

In the hospitality sector, employees often face high stress and interpersonal challenges. Frontline employees with higher levels of hope and optimism are more likely to respond constructively to hazing by seeking support or reframing the experience. An optimistic employee may perceive hazing as a chance to build resilience and seek mentorship, as opposed to feeling victimized (Khliefat et al., 2021; Bellamkonda & Pattusamy, 2024), highlighting the moderating role of hope and optimism in workplace stress dynamics. Research has shown that psychological capital, which includes hope and optimism, enhances mental health and reduces perceptions of job insecurity and stress. These traits help employees maintain a positive outlook and engage in proactive coping strategies, even in the face of workplace hazing.

To mitigate the effects of workplace hazing, organizations can implement interventions that foster hope and optimism among employees. Supportive leadership. Leaders can promote a positive work environment by recognizing and reinforcing hopeful and optimistic behaviors (Khliefat et al., 2021). Training programs. Offering training that focuses on building psychological capital can help employees develop resilience, hope, and optimism. Mentorship and support networks. Establishing mentorship programs and support networks can provide employees with the resources and encouragement they need to cope with stressors constructively. Employees who maintain an optimistic outlook are better equipped to handle stress, which, in turn, reinforces their positive mindset. This creates a feedback loop that enhances overall well-being and reduces the likelihood of deviant behaviors resulting from emotional exhaustion (Alwheshi et al., 2024; Jia et al., 2022). Therefore, this study hypothesizes that hope and optimism moderate the relationship between workplace hazing and emotional exhaustion. Drawing from the arguments presented, the study hypothesizes that

**H5.**  *Hope and optimism moderate the positive relationship between workplace hazing and emotional exhaustion, such that the positive relationship is weaker at a high level of hope and optimism.*

**H6.**  *Hope and optimism moderate the positive relationship between workplace hazing and deviant behavior, such that the positive relationship is weaker at a high level of hope and optimism.*

**H7.**  *Hope and optimism moderate the indirect relationship between workplace hazing and deviant behavior through emotional exhaustion, such that the positive relationship is weaker for employees with a high level of hope and optimism.*

The conceptual model for this study integrates transactional stress theory and examines the relationship between workplace hazing, emotional exhaustion, deviant behavior, and the moderating role of hope and optimism. Workplace hazing serves as the primary stressor (independent variable) leading to emotional exhaustion, which acts as the mediating variable linking hazing to deviant behavior (dependent variable). Emotional exhaustion, a key dimension of burnout, represents the psychological strain employees experience due to workplace stressors. The model further posits that hope and optimism act as a moderating variable, buffering the impact of workplace hazing on emotional exhaustion and subsequently mitigating the effects of emotional exhaustion on deviant behaviour. Nielsen et al. (2024) highlights that workplace bullying is similar to hazing and leads to emotional exhaustion as employees struggle with toxic environments. Similarly, another study (Chiyangwa & Muponya, 2024) identified emotional exhaustion as a significant predictor of deviant behavior in individuals with diminished coping resources. This conceptual framework provides a robust basis for understanding the mechanisms and mitigating factors underlying the relationship between workplace hazing and deviant behavior.

## 3. Research Methodology

### 3.1. Research Design

We confirmed with the supervisors and employees of various five-star hotels whether they would be willing to participate in our research. The hotel management that accepted our invitations was then mailed a pair of questionnaire surveys (with matching return envelopes). The surveyed hotel management communicated the objectives of the study to the respondents to address any concerns they might have and the process involved in completing the survey. This study adopts the approach of administering the questionnaires to multiple sources. To this end, in the first wave, the questionnaires were to the employees. The hotel employees completed the questions related to demographic characteristics, workplace hazing, emotional exhaustion, and hope and optimism. In the second wave, the data were collected from hotel supervisors (a month after the first wave). The supervisors were asked to rate the deviant behavior of the hotel employees. Finally, 800 matching questionnaires were administered in the current study (one supervisor overseeing 10 employees). Furthermore, each respondent was given a unique identifier (i.e., a code) that is consistent across the two waves.

### 3.2. Sampling and Data Collection

This study collected data from employees and supervisors working in four- and five-star hotels in Aqaba, Amman, and Petra in Jordan. We chose the hotel sector as this study’s research context because the sector has been reported as a hostile work environment where employees are often subjected to stressful interactions and abusive practices with their supervisors (Khalid et al., 2024; Srivastava et al., 2024). Moreover, for the hospitality sector to stay competitive, they must focus on their employees (Breitsohl & Garrod, 2016). Hence, data were collected from employees and supervisors of four and five-star hotels in Jordan.

Data were collected using the convenience sampling method. Congruent with the literature, we adopted convenience sampling, a non-probabilistic sampling approach to obtain offline data from the hotel sector. The employees and supervisors were approached following management’s approval. Questionnaires were administered to the targeted respondents who expressed willingness to participate. The questionnaires, which were originally developed in English, were translated to Arabic by using the expert approach of (Brislin, 1970) back-translation. Furthermore, we explained to the targeted respondents to ensure the completion of the questionnaires due to different time intervals.

The selection process considered 27 four- and five-star hotels, which collectively employed 2133 individuals. We then used the widely known Slovin formula for suitable sample size determination to determine the appropriate sample size for our study. This widely known formula was recently used by Aboalhool et al. (2024).

$$n=\frac{N}{1+{N(e)}^{2}}$$

$$n=\frac{2133}{{\left(\{1+2133*0.05\right)}^{2}}$$

N = 337

A total number of 800 questionnaires were administered, out of which 567 were recovered. However, after removing incomplete responses, 494 valid responses were obtained, resulting in a response rate of 61.75%. Table 1 illustrates the information of the targeted respondents.

Based on gender, males account for 257 (52.02%) and females account for 237(47.98). As per the decision of the respondents, bachelor’s degrees account for 381 (77.13%), master’s degrees, 77 (15.58%), doctoral degrees 5 (1.01%), and others 31 (6.28%). Based on experience, less than 3 years account for 46 (9.31%); 3–6 years, 168 (34.01%); 7–10 years, (50.81%); and more than 10 years, 29 (5.87%).

### 3.3. Measures

Workplace hazing was measured with 15 items adopted from (Mawritz et al., 2022). An item was “Verbally humiliated me”. Emotional exhaustion was measured with 3 items adopted from (Boswell et al., 2004). One item was “I feel burned out from my work”. Hope and optimism were measured with 6 items adopted from (Grobler & Joubert, 2018). One item was “When things are uncertain for me at work, I usually expect the best”. Deviant behavior was measured with 10 items adopted from (Bennett & Robinson, 2000). An item was “Intentionally worked slower than you could have worked”. Appendix A Table A1 presents the measurement items for analysis.

### 3.4. Analytical Procedures

The respondents’ demographic information was tabulated using descriptive analysis. To analyze the link among WH, EE, HO, and DB, bivariate correlation was used. Before testing the proposed framework, the measurement model was examined using CFA.

Using the PROCESS macro for SPSS, the researchers examined the direct, mediation, and moderation analyses (Hayes, 2018). This bias-corrected 95% confidence interval was calculated using 5000 bootstrap resample. Model 4 of the PROCESS macro was utilized to test the direct effects and determine whether the link among WH and DB was mediated by EE. The moderation effects were subsequently tested using model 15 of the PROCESS macro. A conditional direct and indirect effect can be established if the 95% confidence interval of the interactions does not contain zero.

Additionally, the conditional direct effects were visualized to obtain a comprehensive understanding of the interactions, by the simple slope test procedure established by (Aiken, 1991), which was recently used by (Abuzawida et al., 2023). Covariates, including gender and education, were added to the moderation analyses. Statistical analyses were conducted using SPSS 29 and AMOS 26.

## 4. Data Analysis and Results

### 4.1. Non-Response Bias

We examined the sample collected for non-response bias through a *t*-test that assessed the differences between early and late respondents. Out of the 494 valid responses obtained, approximately 81% (399 participants) were received within the first month of the period of the survey, compared with 19% (95 participants) received in the second month. To conduct the non-response bias test, we grouped the survey received into two “waves” based on the return dates, with the late respondents serving as the representatives of those who did not respond (Lambert & Harrington, 1990). The results showed no significant difference between the early and the late respondents (*p* > 0.05). Hence, non-response bias is not a concern in this study.

### 4.2. Common Method Bias (CMB)

Method bias represents a source of measurement error that may lead to incorrect links among the items being measured. Systematic error variance can influence research findings, leading to inaccurate outcomes. Therefore, it is important to check for CMB in survey research. Following organizational studies (Al Tera et al., 2024; Alwheshi et al., 2024) and the recommendation of (Tehseen et al., 2017), both procedural and statistical measures were adopted. To this end, the items were refined using a three-thorough procedural remedy. They involved clarifying item definitions, ensuring the specificity and simplicity of the questions, defining each point on the response scale to minimize ambiguity, and using negatively and positively worded items to deal with acquiescence and dis-acquiescence biases (Podsakoff et al., 2012). In addition, conceptually related variables were arranged at a distance to minimize the consistency tendency of the survey participants (Podsakoff & Organ, 1986).

Furthermore, the statistical approach involved using Harman’s first single-factor test by loading the entire measurement items into exploratory factor analysis (Podsakoff et al., 2003). The results indicate that of the entire four factors with eigenvalue greater than 1.0, the first factor only explained 27.76% of the total variance, indicating CMB is not a serious concern in this study. Additionally, the correlation procedure was used, using a theoretically unrelated variable as a marker variable (Lindell & Whitney, 2001). The results showed that the unrelated variable had a correlation of less than 0.05 with the main variable of the study. In sum, the results of the statistical remedies demonstrate that CMB is not a serious issue in this study.

### 4.3. Measurement Model Validation

CFA procedures were employed to test the reliability of the model (Hair et al., 2010). Table 2 presents the reliability and validity of the measurement model. The results indicate that the measurement items consistently aligned with the constructs they measure with Cronbach’s alpha value higher than 0.8, indicating a strong level of reliability (Fornell & Larcker, 1981). Furthermore, skewness and kurtosis values (demonstrated in Table 2) were within the recommended range |2|, |3|, respectively, showing normal distribution of the sample data (Lei & Lomax, 2005).

**Figure 2:**
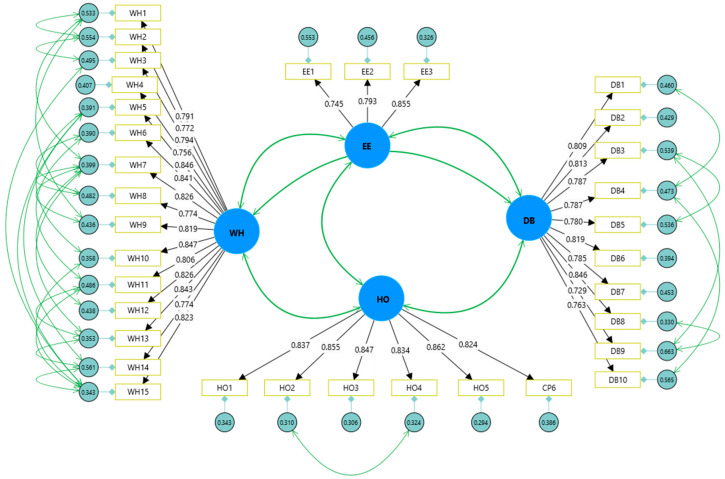
Loadings of the measurement items.

The CFA results revealed that the factor loadings of all theoretical constructs were higher than 0.7 (between 0.729 and 0.862) in Table 2 and Figure 2, demonstrating the convergent validity of the constructs (Fornell & Larcker, 1981). Furthermore, the CR was above 0.7 (between 0.838 and 0.966) and AVE values were higher than 0.5 (between 0.628 and 0.711). Consistent with the results, the theoretical constructs of this study have satisfactory convergent validity (Fornell & Larcker, 1981).

Furthermore, we computed discriminant validity by comparing the correlations between theoretical constructs and the square root of AVEs (Fornell & Larcker, 1981). Table 3 presents that the square root of AVEs (of all constructs) was higher than the correlations between any pair of constructs, offering support for discriminant validity. Additionally, the correlation between all pairs of constructs was 0.8 and the VIF was less than 5, indicating no multicollinearity issue (Hair, 1998; Iyiola & Rjoub, 2020).

Finally, Table 4 indicates that RFI, CFI, IFI, NFI, and TLI were above 0.9, GFI was above 0.8, RMSEA was below the recommended threshold of 0.05, and the CMIN/df also had a value of 2.716, which is below the recommended value of 3, indicating the research model shows a good fit with the data (Hair et al., 2010).

### 4.4. Mediation Model

The proposed mediation model (direct and indirect effect) was tested using Model number 4 of the (Hayes, 2018) PROCESS macro. We adhered to the four-step procedure (Baron & Kenny, 1986) to examine the direct and indirect effects: the first step involves WH significantly predicting DB; the second step involves WH significantly predicting EE; the third step involves emotional exhaustion significantly predicting DB. With the inclusion of the mediator (i.e., emotional exhaustion), if the direct effect remains significant, a partial mediating effect is observed. Table 5 presents the results of the mediation model. In Model 1, WH significantly and positively predicts EE (β = 0.825, t = 28.991, *p* < 0.001). WH significantly and positively predicts DB (β = 0.500, t = 16.248, *p* < 0.001). WH significantly and positively predicts DB (β = 0.500, t = 16.248, *p* < 0.001). EE significantly and positively predicts DB (β = 0.438, t = 14.698, *p* < 0.001). Based on these results, H1–H3 were supported. Table 5 illustrates that with the inclusion of EE as a mediator, the direct effect remains significant. Hence, EE partially mediates the link between WH and DB.

To provide the most reliable results for the mediation analysis. We employed the bootstrapping technique with 5000 resamples. The bias-corrected bootstrap mediation analysis confirms that EE partially mediates the direct effect. Table 5 shows that the indirect effect of EE was 0.361 and the 95% CI [0.287, 0.432]. Zero excludes the lower bound and the upper bound of the confidence interval, confirming that EE partially mediates the link between WH and DB. Hence, H4 is supported.

### 4.5. Moderated Mediation Model

The proposed moderated mediation model (conditional direct and indirect effect) was tested using Model number 59 of the (Hayes, 2018) PROCESS macro. Following recent organizational studies (Iyiola et al., 2023), (Ramadhan et al., 2024), we mean-centered the constructs before testing the interaction effects to prevent multicollinearity problems. In the model, gender and education were included as covariates. Table 6 presents the results of the moderation model.

**Figure 3:**
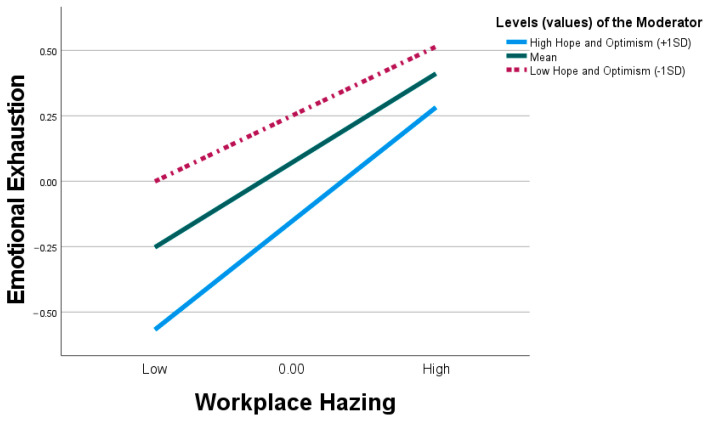
Interaction between workplace hazing and hope and optimism on emotional exhaustion.

Table 6 in Model 1 shows that WH significantly predicts emotional exhaustion (β = 0.343, t = 6.874, *p* < 0.001), with the relationship moderated by the interaction between WH and HO (β = 0.100, t = 4.883, *p* < 0.001, [0.060, 0.141]). We clarified the interpretation of the interaction effect by plotting the predicted EE by WH high and low levels of HO using the simple slope test recommended by (Aiken, 1991). The simple test depicts (Figure 3) that for high HO individuals, the effect of WH on EE was weaker (β = 0.265 t = 4.779, *p* < 0.001, [0.157, 0.376]). However, for individuals with low HO, the effect of WH on EE was more pronounced (i.e., stronger) (β = 0.439, t = 8.746, *p* < 0.001, [0.341, 0.538]). Based on these results, we found support for H5.

**Figure 4:**
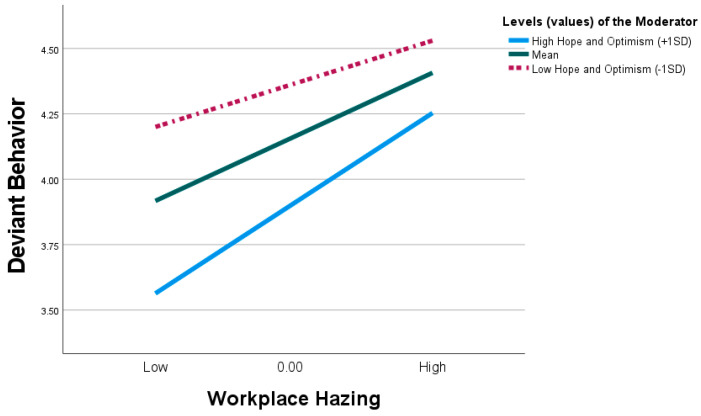
Interaction between workplace hazing and hope and optimism on deviant behavior.

Table 6 in Model 2 indicates that WH significantly predicts DB (β = 0.253, t = 7.521, *p* < 0.001), with the relationship moderated by the interaction between WH and HO (β = 0.108, t = 3.202, *p* ≤ 0.001, [0.042, 0.174]). The simple test indicates (Figure 4) that for high HO individuals, the effect of WH on DB was less pronounced (i.e., weaker) (β = 0.171, t = 3.951, *p* ≤ 0.001, [0.086, 0.256]). Whereas, for individuals with low HO, the effect of WH on EE was more pronounced (i.e., stronger) (β = 0.356, t = 7.796, *p* < 0.001, [0.267, 0.447]). Based on these results, we found support for H6.

**Figure 5:**
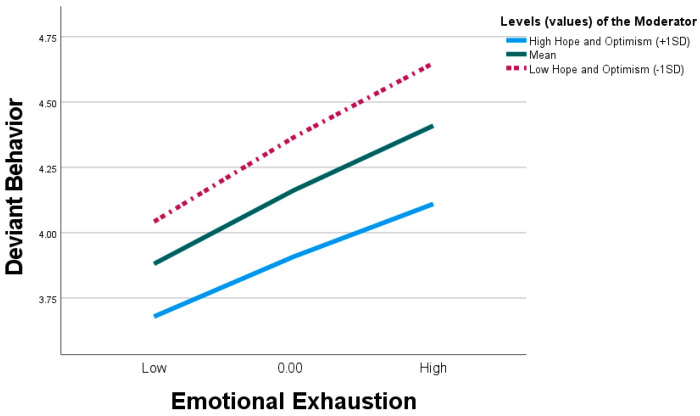
Hope and optimism moderate the indirect effect of interaction between workplace hazing on emotional exhaustion.

In addition, testing the conditional indirect effect of WH on DB through EE at varying levels of the moderator (i.e., values of the moderator: +1 SD, mean, and −1 SD) were all significant, indicating that HO moderates the indirect effect of WH on DB through EE. Figure 5 shows that the indirect effect of WH on DB through EE was significant for both high (β = 0.086, [0.040, 0.144]) and low (β = 0.101, [0.055, 0.155]) levels of optimism. The result indicates a weaker link between WH DB through EE in individuals with high HO. Hence, we found support for H7.

## 5. Discussion

The findings of this study provide a nuanced understanding of the intricate relationships between workplace hazing, emotional exhaustion, and deviant behavior within the hospitality sector, supported by the transactional stress theory framework. By examining the mediating role of emotional exhaustion and the moderating effects of hope and optimism, this research contributes valuable insights into how psychological and environmental factors interact to influence employee behavior. These results underscore the critical need for organizational strategies that mitigate workplace hazing, address emotional exhaustion, and foster resilience through positive psychological interventions, promoting healthier and more productive workplace cultures.

According to transactional stress theory, hope and optimism play a crucial role in cognitive appraisal, significantly influencing how an individual perceives and copes with a stressful situation (Si et al., 2023). These factors promote a positive outlook and belief in one’s ability to manage challenges, thereby leading to more effective coping mechanisms and resilience in the face of adversity (Mikus & Teoh, 2022). To explore the impact of workplace hazing on deviant behavior in the hospitality sector with a focus on the mediation role of emotion exhaustion and the moderation effect of hope and optimism. The hypothesis test reflects these relationships and provides meaningful insights into workplace hazing and deviant behavior in the hospitality sector.

In this study, workplace hazing and emotional exhaustion confirmed a significant positive link between workplace hazing and deviant behavior. This evaluation might even be undervalued because most studies (Sarwar & Muhammad, 2020) have focused on understanding the severe forms and organizational impacts of deviant behavior. Those who experienced hazing were more engaged in deviant acts such as absenteeism and lateness. This suggests that hazing creates a toxic environment that encourages employees to rebel against organizational norms, which, in turn, leads to deviant behavior. Additionally, previous scholars have already demonstrated a positive relationship between workplace hazing and deviant behavior (Jiang et al., 2021). The results confirmed that workplace hazing significantly contributes to emotional exhaustion. The employees who experienced hazing expressed a sense of mental and emotional exhaustion, a sentiment that is consistent with the previous research. Hazing adds another layer of stress and depletes emotional resources in situations where employees already face demanding workloads, leading to burnout (Shaikh & Usmani, 2022).

Emotional exhaustion and deviant behavior. This result confirms the positive link. The results align with prior research emphasizing the adverse effects (Kong et al., 2020; Jahanzeb & Fatima, 2017). Severe forms of deviant behavior have been widely studied by Kong et al. (2020) (Sarwar et al., 2021). Employees who experience a high level of emotional exhaustion are more likely to engage in deviant behavior. As a result of emotional exhaustion, employees may engage in counterproductive work behavior as a form of response to their inability to cope with workplace demands (Ul Haq & Huo, 2024). This research employed the bootstrapping technique with 5000 resamples. The bias-corrected bootstrap mediation analysis confirms that emotional exhaustion plays a part in mediating the direct effect. This means that hazing may cause burnout, which, in turn, causes deviant behavior. This mediation role highlights the critical role of emotional exhaustion in a positive workplace (Kumar & Jin, 2022). This research makes a significant contribution by discovering that hope and optimism moderate the relationship between workplace hazing and emotional exhaustion. Hope and optimism moderate a weaker link between workplace hazing and deviant behavior through emotional exhaustion, particularly in individuals with high levels of hope and optimism (Alwheshi et al., 2024; Jia et al., 2022). Therefore, we found support for H7, which posits an indirect relationship between workplace hazing and deviant behavior via emotional exhaustion. This finding suggests that fostering a positive psychological environment among employees and promoting resilience can help mitigate the harmful effects of workplace hazing. By enhancing hope and optimism, organizations have provided employees with the emotional resources needed to cope with workplace stressors, reducing the likelihood of deviant behavior (Hao et al., 2015; Jiang et al., 2021).

## 6. Conclusions

### 6.1. Theoretical Contribution

This research makes several theoretical contributions. Firstly, the study utilized the transaction theory of stress to elucidate the link between workplace hazing and deviant behavior. The transaction theory of stress, which emphasizes the interaction between the individual and the environment through cognitive appraisal, helps explain how employees who perceive hazing as a threat to their well-being may experience stress, which manifests as deviant behavior. This underscores the significance of cognitive appraisal in understanding workplace hazing and deviant behavior (Ahmed et al., 2024). According to the transaction theory of stress, emotional exhaustion arises when coping resources are insufficient to meet perceived demands. Hazing acts as a chronic stressor, and when appraised as harmful, it leads to emotional exhaustion (Ali et al., 2022). This explains why emotional exhaustion is prevalent in environments with frequent hazing practices, especially in high-pressure sectors like hospitality.

The study demonstrates that emotional exhaustion mediates the link between workplace hazing and deviant behavior. Emotional exhaustion, a key outcome of stress, leads to disengagement among employees, hindering their ability to cope effectively. The theory suggests that emotional exhaustion, which plays a central role in transforming the negative effects of hazing into deviant behavior, mediates the impact of workplace stressors like hazing. To our knowledge, only a few studies have directly examined the mediating role of emotional exhaustion in the link between workplace hazing and deviant behavior (Dodanwala & Shrestha, 2021; Ul Haq & Huo, 2024). This paper is the first to apply the transaction theory of stress approach to the workplace hazing domain to explore how workplace hazing relates to deviant behavior. The transaction theory of stress focuses on the direct effect of stressors and individual coping mechanisms, but this study adds nuance by showing that emotional exhaustion is not just an outcome of stress but also a critical pathway that links the stressor of hazing to negative behavior. Additionally, our findings align with previous studies that confirm the link between workplace hazing and emotional exhaustion (Choi, 2019; Jahanzeb & Fatima, 2017), as well as the association between emotional exhaustion and deviant behavior (e.g., pp. 17, 14). Positive psychological traits such as hope and optimism influence how employees appraise and cope with stress. These traits serve as coping resources, acting as a buffer against the negative effects of work-related stress. Employees with high levels of hope and optimism may perceive hazing as less threatening and cope more effectively, thereby reducing emotional exhaustion and mitigating its impact on deviant behavior. These findings not only address the question of how employees experiencing different levels of emotional exhaustion respond to hazing but also contribute to the development of the transaction theory of stress. To our knowledge, this is the first study to integrate emotional exhaustion, hope and optimism, and deviant behavior with workplace hazing, thereby expanding the literature on workplace hazing.

### 6.2. Practical and Managerial Implications

Managing hazing to reduce deviant behavior. Organizations, particularly in the hospitality sector, should implement clear anti-hazing policies and cultivate a culture that discourages hazing practices. By reducing hazing, the organization can reduce workplace stress and prevent employees from becoming deviant in their behavior (Ahmed et al., 2024). Given that hazing is a contributing factor, emotional exhaustion organizations need to offer mental health support, such as counseling services. Additionally, improving communication and work–life balance and offering more control over the work process can reduce emotional exhaustion. The mediating effect of emotional exhaustion implies that early intervention to reduce emotional exhaustion could prevent employees from exhibiting deviant behavior. This can be carried out, for example, by emphasizing the importance and value of subordinates in organizations and showing more concern for employees. By making employees feel that they are valued and respected, their emotional exhaustion symptoms would be alleviated (Jiang et al., 2021). To prevent emotional exhaustion from leading to deviant behavior, organizations should foster resilience. Building a workshop can help employees develop problem-focused coping strategies, enabling them to better manage emotional exhaustion before it leads to harmful behaviors.

Managers should recognize the early signs of emotional exhaustion as a mediator between workplace hazing and deviant behavior. By implementing regular employee wellness checks and providing preventive mental health interventions, managers can address the issue of emotional exhaustion becoming a driver of a negative work environment. Developing hope and optimism among employees can mitigate the effects of workplace stressors. Training programs emphasize positive psychology, mentorship, and leadership to foster an optimistic outlook, thereby enhancing the employee’s ability to cope with stress. By fostering these psychological resources, we can reduce employee stress and the likelihood of deviant behavior in the hospitality sector (Chiyangwa & Muponya, 2024).

### 6.3. Limitations and Future Studies

This study provides valuable insights into the role of workplace hazing in influencing deviant behavior in the hospitality sector; however, several limitations need to be addressed in future research. First, the study employed convenience sampling, which may limit the generalizability of the findings to broader populations. Future studies could use probability sampling to enhance representativeness.

Second, the cross-sectional research design restricts the ability to infer causal relationships among variables. Longitudinal studies would provide a more comprehensive understanding of the dynamics between workplace hazing, emotional exhaustion, and deviant behavior. While focusing on the hospitality industry’s valuable insights, the findings may not be generalizable to other industries with different workplace dynamics, organizational culture, and stressors.

Additionally, the study focused solely on four- and five-star hotels in Jordan, potentially overlooking industry-specific variations in other regions or types of hospitality establishments. Expanding the geographical scope and including different hospitality contexts could enrich the findings. Furthermore, while hope and optimism were examined as moderators, future research could explore additional psychological and organizational factors, such as resilience or workplace culture, that may mitigate the adverse effects of hazing.

Finally, the reliance on self-reported measures introduces potential biases, including social desirability and recall errors. Incorporating mixed-method approaches, such as qualitative interviews or behavioral observations, would provide a deeper understanding of the phenomenon. Addressing these limitations will enhance the robustness of findings and contribute to the development of effective strategies for mitigating workplace hazing and fostering a supportive work environment.

## Figures and Tables

**Table 1:** Characteristics of the respondents.

Demographic Information of the Respondents (*n* = 494)			
Gender			
Male	257 (52.02%)	Female	237 (47.98%)
Education			
Bachelor	Master	Doctoral degree	Others
381 (77.13%)	77 (15.58%)	5 (1.01%)	31 (6.28%)
Experience			
Less than 3 years	3–6 years	7–10 years	Above 10 years
46 (9.31%)	168 (34.01%)	251 (50.81%)	29 (5.87%)
Position			
Employees	443 (89.68%)	Managers	51 (10.32%)
Hotel Ranking			
Four-star hotels	257 (52.02%)	Five-star hotels	237 (47.98%)

**Table 2:** Confirmatory factor analysis results.

Variables	Item Codes	SFL	CA	CR	AVE	Skewness	Kurtosis
Workplace Hazing			0.966	0.966	0.656		
	WH1	0.791				−1.645	2.812
	WH2	0.772				−1.593	2.771
	WH3	0.794				−1.491	1.751
	WH4	0.756				−1.080	2.508
	WH5	0.846				−1.732	2.375
	WH6	0.841				−1.721	2.215
	WH7	0.826				−1.836	2.265
	WH8	0.774				−1.733	2.939
	WH9	0.819				−1.733	2.759
	WH10	0.847				−1.953	2.314
	WH11	0.806				−1.495	2.336
	WH12	0.826				−1.693	2.100
	WH13	0.843				−1.308	2.921
	WH14	0.774				−1.854	2.398
	WH15	0.823				−1.433	2.335
Emotional Exhaustion			0.838	0.841	0.638		
	EE1	0.745				−1.009	2.404
	EE2	0.793				−1.062	2.828
	EE3	0.855				−1.363	2.228
Hope and Optimism			0.935	0.937	0.711		
	HO1	0.837				−1.677	1.945
	HO2	0.855				−1.517	2.241
	HO3	0.837				−1.675	2.349
	HO4	0.824				−1.715	2.622
	HO5	0.862				−1.627	2.763
	HO6	0.824				−1.462	2.669
Deviant Behavior			0.944	0.944	0.628		
	DB1	0.809				−1.968	1.114
	DB2	0.813				−1.143	2.021
	DB3	0.787				−1.028	2.077
	DB4	0.787				−1.168	2.216
	DB5	0.780				−1.051	1.275
	DB6	0.819				−1.425	1.486
	DB7	0.785				−1.240	2.771
	DB8	0.846				−1.359	2.466
	DB9	0.729				−1.820	2.230
	DB10	0.763				−1.023	2.258

Note: CA = Cronbach’s alpha, CR = composite reliability, AVE = average variance extracted, SFL = standard factor loading, WH = workplace hazing, EE = emotional exhaustion, HO = hope and optimism, DB = deviant behavior.

**Table 3:** Sample’s descriptive statistics, correlation, and discriminant validity.

Constructs	Mean	STD	Workplace Hazing	Emotional Exhaustion	Hope and Optimism	Deviant Behavior	Gen	Edu
Workplace hazing	4.031	0.938	(0.810)					
Emotional exhaustion	4.139	0.964	0.584	(0.799)				
Hope and optimism	4.150	0.942	0.629	0.553	(0.843)			
Deviant behavior	4.134	0.929	0.462	0.451	0.606	(0.792)		
Gen	1.358	0.480	−0.007	0.005	−0.013	0.044	-	
Edu	2.469	1.513	−0.018	−0.032	−0.022	−0.029	−0.312	-

Note: STD = standard deviation, Gen = gender, Edu = education.

**Table 4:** Model fitness.

Indices	Suggested Cut-Offs	Results
CMIN/df	<3	1347.001/496 = 2.716
GFI	>0.8	0.860
RMSEA	<0.08	0.060
RFI	>0.9	0.907
TLI	>0.9	0.939
NFI	>0.9	0.917
IFI	>0.9	0.946
CFI	>0.9	0.946

**Table 5:** Hypotheses testing: Mediation model (direct and indirect effects).

Outcome Construct	Independent Variable	β	S. E	T	*p*	95% CI
Model 1: Emotional exhaustion	Intercept	0.811	0.118	6.906	<0.001	[0.580, 1.042]
	Workplace hazing	0.825	0.028	28.991	<0.001	[0.767, 0.880]
R^2^ = 0.537						
Model 2: Deviant behavior	Intercept	0.305	0.080	3.806	<0.001	[0.148, 0.463]
	Workplace hazing	0.500	0.031	16.248	<0.001	[0.440, 0.561]
	Emotional exhaustion	0.438	0.030	14.698	<0.001	[0.380, 0.497]
R^2^ = 0.712						
The indirect effect of workplace hazing on deviant behavior via emotional exhaustion						
	Direct effect of X on Y	0.500	0.031	16.248	<0.001	[0.440, 0.561]
Bootstrap indirect effects			BootSE		BootLLCI	BootULCI
Workplace hazing → Emotional exhaustion → Deviant behavior		0.361	0.037		0.287	0.432

Note: CI = confidence interval, BootLLCI = bootstrap lower level of confidence interval, BootULCI = bootstrap upper level of confidence interval; standard error.

**Table 6:** Hypotheses testing: Conditional direct and indirect effects (moderation analyses).

	β	S. E	t	*p*	95% CI
Model 1: Predicting emotional exhaustion					
Intercept	0.080	0.029	2.708	0.007	[0.022, 0.137]
Gender	−0.017	0.054	−0.206	0.837	[−0.095, 0.117]
Education	0.030	0.017	0.173	0.863	[−0.031, 0.037]
Workplace hazing	0.343	0.050	6.874	0.000	[0.246, 0.443]
Emotional exhaustion	0.230	0.075	3.063	0.023	[0.083, 0.378]
Workplace hazing × emotional exhaustion	0.100	0.021	4.883	0.000	[0.060, 0.141]
R^2^ = 0.715					
The conditional direct effect of workplace hazing on emotional exhaustion at different levels of hope and optimism					
Hope and optimism (−1 SD)	0.439	0.050	8.746	0.000	[0.341, 0.538]
Hope and optimism (M)	0.343	0.050	6.816	0.000	[0.244, 0.442]
Hope and optimism (+1 SD)	0.265	0.056	4.779	0.000	[0.157, 0.376]
Model 2: Response variable model for predicting deviant behavior					
Constant	4.153	0.063	65.690	0.000	[4.029, 4.278]
Gender	−0.004	0.034	−0.120	0.905	[−0.072, 0.064]
Education	0.006	0.011	0.542	0.588	[−0.016, 0.027]
Workplace hazing	0.253	0.034	7.521	0.000	[0.187, 0.320]
Emotional exhaustion	0.282	0.030	9.478	0.000	[0.223, 0.340]
Hope and optimism	0.265	0.049	5.445	0.000	[0.169, 0.360]
Workplace hazing × hope and optimism	0.108	0.034	3.202	0.001	[0.042, 0.174]
Emotional exhaustion × hope and optimism	0.054	0.033	1.674	0.095	[−0.09, 0.118]
The conditional direct effect of workplace hazing on deviant behavior at different levels of hope and optimism					
Hope and optimism (−1 SD)	0.356	0.046	7.796	0.000	[0.267, 0.447]
Hope and optimism (M)	0.253	0.034	7.521	0.000	[0.187, 0.320]
Hope and optimism (+1 SD)	0.171	0.043	3.951	0.001	[0.086, 0.256]
The conditional indirect effect of X on Y: The conditional indirect effect of workplace hazing on deviant behavior through emotional exhaustion at different levels of hope and optimism					
Hope and optimism (−1 SD)	0.101	0.025			[0.055, 0.155]
Hope and optimism (M)	0.097	0.024			[0.55, 0.147]
Hope and optimism (+1 SD)	0.086	0.026			[0.040, 0.144]

Note: CI = confidence interval.

## Data Availability

The data from this study can be requested from the conservation of resources theory corresponding author, Ahmad Alzubi.

## Appendix A

**Table A1:** Measurement items.

**Workplace Hazing (Mawritz et al., 2022)**
1. Segregated me from our work group
2. Excluded me from our work group
3. Refrained from socializing with me.
4. Ridiculed me.
5. Verbally humiliated me.
6. Verbally embarrassed me.
7. Directed me to work on tasks that are not relevant to the future work I will do for the group.
8. Given me unimportant tasks to complete.
9. Withheld useful information from me about how to accomplish tasks.
10. Physically harmed me.
11. Deprived me of food.
12. Gotten physically aggressive with me (shoving, slapping, hitting).
13. Told me stories that are untrue to see how naive I am.
14. Played pranks on me to test my gullibility.
15. Told me lies to see if I am a pushover.
**Emotional exhaustion (Boswell et al., 2004)**
1. I feel emotionally drained from my work
2. I feel burned out from my work.
3. I feel exhausted when I think about having to face another day on the job.
**Hope and optimism (Grobler & Joubert, 2018)**
1. At the present time, I am energetically pursuing my work goals.
2. Right now, I see myself as being pretty successful at work.
3. I can think of many ways to reach my current work goals.
4. At this time, I am meeting the work goals that I have set for myself
5. When things are uncertain for me at work, I usually expect the best.
6. I always look on the bright side of things regarding my job
**Deviant behavior (Bennett & Robinson, 2000)**
1. Spent too much time fantasizing or daydreaming instead of working.
2. Falsified a receipt to get reimbursed for more money than you spent on business expenses.
3. Taken an additional or longer break than is acceptable at your workplace.
4. Come in late to work without permission.
5. Neglected to follow your boss’s instructions.
6. Intentionally worked slower than you could have worked.
7. Discussed confidential company information with an unauthorized person.
8. Used an illegal drug or consumed alcohol on the job.
9. Put little effort into your work.
10. Dragged out work to get overtime.
